# Implications of endophytic microbiota in *Camellia sinensis*: a review on current understanding and future insights

**DOI:** 10.1080/21655979.2020.1816788

**Published:** 2020-09-15

**Authors:** Hengtong Xie, Xiaoxiao Feng, Mengcen Wang, Yuefei Wang, Mukesh Kumar Awasthi, Ping Xu

**Affiliations:** a College of Agriculture and Biotechnology, Zhejiang University, Hangzhou, China; b Agricultural Experiment Station of Zhejiang University, Hangzhou, China; c Key Laboratory of Molecular Biology of Crop Pathogens and Insects, Ministry of Agriculture, Hangzhou, China; d Key Laboratory of Horticultural Plant Growth, Development and Quality Improvement, Ministry of Agriculture, Hangzhou, China; e College of Natural Resources and Environment, Northwest A&F University, Yangling, China

**Keywords:** *Camellia sinensis*, endophytic microbiota, isolation, identification, diversity, biological functions

## Abstract

Endophytic fungi and bacteria are the most ubiquitous and representative commensal members that have been studied so far in various higher plants. Within colonization and interaction with their host plants, endophytic microbiota are reportedly to modulate not only the host’s growth but also holobiont resilience to abiotic and biotic stresses, providing a natural reservoir and a promising solution for sustainable agricultural development challenged by global climate change. Moreover, possessing the talent to produce a wide array of high-value natural products, plant endophytic microbiota also serve as an alternative way for novel drug discovery. In this review, tea, one of the world’s three largest nonalcoholic beverages and a worldwide economic woody crop, was highlighted in the context of endophytic microbiota. We explore the recent studies regarding isolation approaches, distribution characteristics and diversity, and also biological functions of endophytic microbiota in *Camellia sinensis* (L.) O. Kuntze. Profoundly, the future insight into interaction mechanism between endophytic microbiota and tea plants will shed light on in-depth exploration of tea microbial resources.

## Graphic abstract

**Figure:**
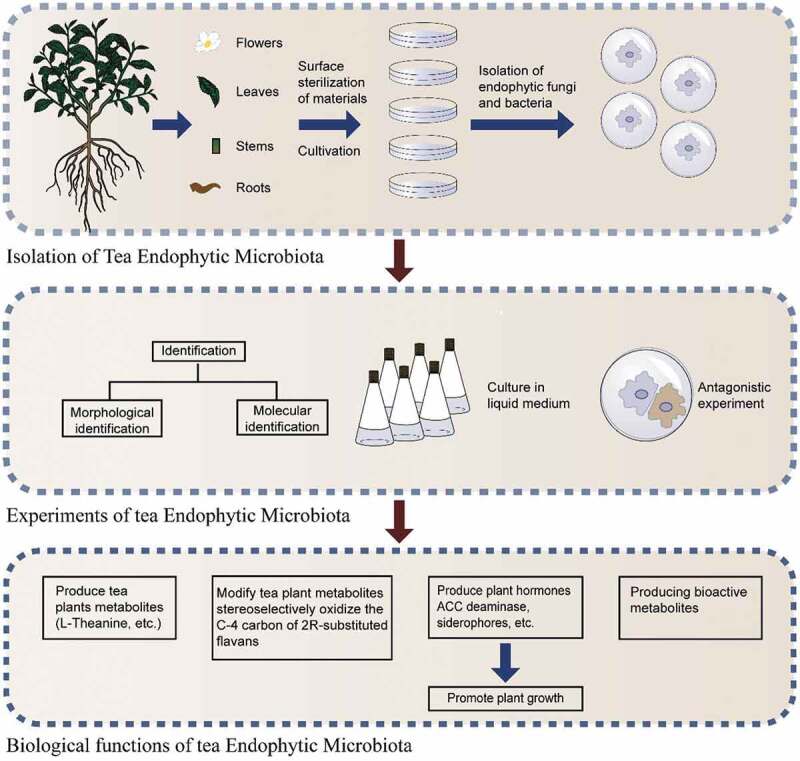

## 1. Introduction

In the past four decades, endophytes have attracted the attention of taxonomists, ecologists, evolutionary biologists, and microbiologists [1]. Endophytes are found in almost vascular plants, host plant organs, and even their seeds [2]. With the deepening of research, endophytic fungi and bacteria are a new type of microbial resources, which have great research value and broad development prospects. Under the long-term symbiotic evolution, endophytes and the host have established a mutually beneficial relationship [3]. On the one hand, endophytes obtain the nutrients needed for growth from the host plant; on the other hand, endophytes confer a beneficial effect on the physiological activities of the host. Typically, some endophytic microbes play important roles in promoting the growth of the host plant by producing biological active molecules, such as indoleacetic acid, gibberellin, cytokinin, siderophores, phosphate-solubilizing enzymes and 1-aminocyclopropane-1-carboxylate (ACC) deaminase [4]. Besides, some nitrogen-fixing bacteria can improve soil fertility through nitrogen fixation to promote growth [5,6]. Utilization of these interactions between plants and endophytes opens a door to improve the crop productivity through manipulation of the plant growth promotion effect [7].

In addition, endophytic microbiota were also reportedly to modulate host plants’ resistance to abiotic and biotic stress [8]. Endophytic fungi and bacteria can reduce the incidence of disease dominantly via antagonizing phytopathogens by nutrient and space competition [9], producing various antagonistic secondary metabolites [10], and inducing the expression of host defense-related genes [9]. Therefore, endophytic fungi and bacteria are promising reservoirs for screening of candidates for development of biological control agents [11]. Abiotic stress has a negative effect on plant physiology and morphology [12], endophytic fungi can help their host plants adapt to abiotic stress and promote plant growth through plant hormone biosynthesis and nutrient absorption [13]. For instance, the discovery of the mycelium and spores of fossil fungi indicates that endophytic fungi are essential for the resistance of land plant ancestors to abiotic stresses (such as drought, salinity, metals, ultraviolet radiation) [14].

Biotechnologically, endophytic microbiota function as an abundant source for production of various secondary metabolites, including alkaloids, terpenes, steroids, polyketides, quinones, iso-coumarins, esters, flavonoids, and lactones, *et al*. [15,16]. Possessing antibacterial, antifungal, insecticidal, antioxidant, cytotoxic and anticancer properties [17], these microbiota-derived compounds enrich the chemical diversity of biologically active molecules and provide new ways for drug development [18]. More interestingly, some plant endophytic fungi were reported to produce secondary metabolites with high medicinal value, such as paclitaxel, camptothecin, *et al*. [8,18], which provide solutions to the shortage of medical resources and ecological damage caused by the slow growth and shortage of certain medical plants, and can be used as a bioengineering tool for drug production [19].

As one of the world’s three largest nonalcoholic beverages, tea is known as a healthy beverage in the 21st century [20]. Today, nearly half of the world’s people drink tea. Further, tea plant is a worldwide economic woody crop and play essential roles in forest ecosystem irrespective of largely unexploited ecological functions [21]. Carrying out research on endophytes in tea plants provides not only a promising solution for sustainable agricultural development challenged by global climate change, but also an alternative approach in drug discovery. In this review, we explore the recent studies regarding isolation approaches, distribution characteristics and diversity, and also biological functions of endophytic microbiota in *Camellia sinensis* (L.) O. Kuntze. The future insight into interaction mechanism between endophytic microbiota and tea plants will shed light on in-depth exploration of tea microbial resources.

### 1.1. Definition of endophyte

Endophyte is commonly found in various terrestrial and aquatic plants that have been studied so far. Microorganisms have been found in healthy plants as early as 100 years ago. The term endophyte was proposed by Heinrich Anton de Bary in 1884 and was originally defined as any organism present in plant tissue [22]. The current definition of plant endophytes is inconclusive, the most common definition was proposed by Petrini [23]. All organisms inhabiting plant organs that at some time in their life, can colonize internal plant tissues without causing apparent harm to the host”. According to this definition, fungi, bacteria, insects, algae and other vascular plants should be included [14].

## 2. Isolation and identification of tea endophytic fungi and bacteria

### 2.1. Isolation and identification of tea endophytic fungi

Isolation of tea endophytic fungi includes several steps. First, the collected plant materials are placed under running tap water to remove surface impurities. After proper washing, surface sterilization is performed, however, there are many alternatives listed in Table 1. Surface sterilization requires alcohol solution and mercury chloride or sodium hypochlorite solution. Plant tissues are immersed in these solutions at different times. After proper surface sterilization, plant materials are segmented. For leaf tissues, use a sterilized cork borer to remove 6-mm-diameter discs [24] or cut into appropriately sized segments by sterilized blade [25]. For stem tissues, use a sterilized razor blade to separate the bark and xylem, and cut into 3 mm^2^ segments [24] or cut into segments of appropriate length [26]. Also, materials can be put into sterilized distilled water to prepare the stock tissue homogenate [27]. Finally, inoculate those segments or spread the stock tissue homogenate on the medium and place the Petri dishes in the incubator for proper growth of fungal colonies [27,28]. It should be noted that different fungi show preference for different types of culture media. At present, the isolation of tea endophytic fungi mainly uses potato dextrose agar (PDA) medium, which will reduce the diversity of isolated endophytic fungi.

**Table 1.** Protocols for external tissue sterilization for tea endophytic fungi isolation.

Surface Sterilization Procedure	Reference
Immersed the materials in 80% ethanol for 60s, 1% NaClO for 60s, and 80% ethanol for 60s. Then wash the disinfected materials twice in sterilized distilled water for 60s.	[24]
The surface of materials sterilized in 70% ethanol for 1 min, then immersed in 0.5% NaClO for 3 min followed by 70% ethanol for 1 min and rinsed in sterile water.	[25]
All materials surface sterilized by washing with 70% ethanol for 60s, then immersed in 3% NaClO for 3–5 min, and rinsed in 70% ethanol for 30s, followed by washed in sterile distilled water.	[30]
After proper washing, root materials were dipped in 70% ethanol for 5 min, then immersed in 0.1% HgCl_2_ for 1 min. However, stem and leaf materials were washed with 70% alcohol for 5 min and immersed in 0.05% HgCl_2_ for 1 min. Materials were washed in sterilized distilled water to remove the effect of surface sterilizing agents.	[27]

Identification of tea endophytic fungi includes two methods: morphological [24,26] and molecular identification [24,29,30]. The morphological identification of fungi is based on the colony color, conidia, texture, shape, size, and mycelia color are evaluated [24,31], combined with optical microscopy to observe microscopic features such asmycelia, pycnidia, conidia, asci, and ascospores [31]. For molecular identification, the internal transcribed spacer (ITS) region of the rDNA gene is sequence analyzed. Then the sequences are subjected to the BLAST search in the NCBI database and compare them with representative sequences, then perform phylogenetic analysis and determine fungi species [30,32,33]. Amplification of the ITS region was carried out by using the universal eukaryotic primers of ITS1 and ITS4 [27,30,33,34] or primers of ITS5 and ITS4 [24]. Furthermore, the phylogenetic relationships among taxa in the endophytic fungi are investigated based on DNA sequences of multiple genes, are rewriting the history of taxonomy. The phylogenetic tree is constructed by the maximum likelihood method using MEGA according to the Kimura 2-parameter model. The robustness of the tree is evaluated by performing bootstrap analysis based on 1000 resamplings [30,31].

### 2.2. Isolation and identification of tea endophytic bacteria

Tea endophytic bacteria isolation steps are similar to those of tea endophytic fungi. First, the plant materials are rinsed with tap water, followed by surface sterilized. The surface sterilization methods are similar to those of plant materials in the isolation of tea endophytic fungi [35–37]. After proper surface sterilization, the sterilized samples are cut in a sterilized mortar and grinding to homogenate, followed by proper serial dilution with sterilized water [35,38,39]. Then, the diluted sample tissue homogenates are spread on medium and place the Petri dishes in the incubator.

At present, Luria-Bertani (LB), nutrient agar (NA), King’s B (KB) and tryptic soy agar (TSA) mediums are mainly used for the isolation of tea endophytic bacteria. The development or use of more types of culture media will have a positive significance for the diversity of isolated endophytic bacteria. The identification of tea endophytic bacteria is performed on the basis of cell morphology [36,40], physiological, biochemical tests [36] and molecular identification [35,39,40]. The morphological identification determines the strain species based on the growth morphology, size, color of the bacteria and the growth conditions of the colony surface and edges [36,40]. Physiological and biochemical tests are based on the identification of metabolic products produced by different species during bacterial culture, such as (alcohol) metabolism tests, amino acid and protein metabolism tests, organic acid salt and amine salt utilization tests, respiratory enzyme tests, and toxic enzymes tests, BIOLOG microstation, API tests etc. [36,41]. Morphological identification is usually supplemented by physiological and biochemical tests to identify bacteria [41]. For molecular identification, the 16S rDNA of endophytic bacteria is sequenced and perform sequence analysis and phylogenetic analysis to determine the strain species [35,39,40]. The multilocus sequence analysis (MLSA) approach based on the sequence analysis of the housekeeping genes has proven reliable for the bacteria species discrimination and strain identification [42]

## 3. Diversity of tea endophytic fungi and bacteria

Most endophytes originate from the growth environment of their host plants, including rhizosphere microorganisms, fungal spores suspended in the air, and the feeding process of insects and animals [43]. The propagation methods of endophytic fungi and bacteria are mainly horizontal (plant or soil to plant), vertical (parent plant to seed) or mixture of both [44,45]. According to reports, the construction of endophytic fungi and bacteria communities of plant is not random, determined by the factors such as plant habitat, soil type, plant species and environmental microorganisms [46–49]. Tea plants are rich in endophytic fungi and bacteria. Tables 2 and 3 list and classify the tea endophytic fungi and bacteria. During the previously reported, among the strains that have been identified, endophytic fungi cover three phylums, five classes, 14 orders, 24 families, 34 genera, strains of Pleosporales of Dothideomycetes and Diaporthales, Glomerellales, Hypocreales and Xylariales of Sordariomycetes are the dominant species. Endophytic bacteria cover four phylums, seven classes, 13 orders, 24 families and 32 genera, strains of Micrococcales of Actinobacteria, Bacillales of Bacilli, Rhizobiales of Alphaproteobacteria, and Burkholderiales of Betaproteobacteria are the dominant species. Some of the endophytic fungi and bacteria have not been identified and classified. Therefore, the research on tea endophytic fungi and bacteria needs more comprehensive identification to better study the diversity.

**Table 2.** Endophytic fungi isolated from tea plants.

Phylum	Class	Order	Family	Genus	Species	Reference
Ascomycota	Dothideomycetes	Botryosphaeriales	Botryosphaeriaceae	*Botryosphaeria*	*Botryosphaeria dothidea*	[24]
			Phyllostictaceae	*Guignardia*	*Guignardia mangiferae*	[24]
					*Guignardia* sp.	[24]
		Cladosporiales	Cladosporiaceae	*Cladosporium*	*Cladosporium asperulatum*	[24]
		Mycosphaerellales	Mycosphaerellaceae	*Pseudocercospora*	*Pseudocercospora kaki*	[53]
					*Pseudocercospora* sp.	[24]
		Pleosporales	Didymellaceae	*Didymella*	*Peyronellaea glomerata*	[24]
				*Epicoccum*	*Epicoccum nigrum*	[24]
				*Phoma*	*Phoma herbarum*	[24]
				*Stagonosporopsis*	*Stagonosporopsis cucurbitacearum*	[24]
			Didymosphaeriaceae	*Paracamarosporium*	*Paraconiothyrium hawaiiense* *=Microdiplodia hawaiiensis*	[24]
				*Paraphaeosphaeria*	*Paraphaeosphaeria neglecta*	[24]
			Leptosphaeriaceae	*Plenodomus*	*Plenodomus* sp.	[24]
			Phaeosphaeriaceae	*Setophoma*	*Setophoma chromolaena*	[24]
			Pleosporaceae	*Alternaria*	*Alternaria mali*	[24]
					*Alternaria* sp.	[26]
			/	/	*Pleosporales* sp.	[24]
		/	/	/	*Dothideomycetes* sp.	[24]
	Eurotiomycetes	Eurotiales	Aspergillaceae	*Aspergillus*	*Aspergillus fumigatus*	[27,30]
					*Aspergillus niger*	[27,30,50]
					*Aspergillus* sp.	[26,72]
				*Penicillium*	*Penicillium aculeatum*	[30]
					*Penicillium chrysogenum*	[27]
					*Penicillium citrinum*	[30]
					*Penicillium crustosum*	[27]
					*Penicillium oxalicum*	[30]
					*Penicillium sclerotiorum*	[27,30,53]
					*Penicillium* sp.	[26,27,73]
	Saccharomycetes	Saccharomycetales	/	*Candida*	*Candida* sp.	[26]
	Sordariomycetes	Diaporthales	Diaporthaceae	*Diaporthe*	*Diaporthe eres*	[24]
					*Diaporthe nobilis*	[24]
					*Diaporthe pustulata*	[24]
					*Diaporthe sackstonii*	[24]
					*Diaporthe* sp.	[24,29,58,73]
					*Diaporthe amygdali* *=Phomopsis amygdali*	[24]
					*Diaporthe subordinaria* *=Phomopsis subordinaria*	[24]
			Melanconiellaceae	*Melanconiella*	*Melanconiella* sp.	[24]
			Valsaceae	*Phomopsis*	*Phomopsis* sp.	[24]
		Glomerellales	Glomerellaceae	*Colletotrichum*	*Colletotrichum gloeosporioides*	[30]
					*Colletotrichum* sp.	[50]
					*Colletotrichum* spp.	[26]
					*Glomerella cingulata*	[30]
					*Glomerella* sp.	[24]
		Hypocreales	Hypocreaceae	*Trichoderma*	*Trichoderma koningiopsis*	[24]
					*Trichoderma longibrachiatum*	[72]
					*Trichoderma pseudokoningii*	[74]
					*Trichotderma viride*	[26]
					*Trichotderma* sp.	[26]
			Nectriaceae	*Fusarium*	*Fusarium oxysporum*	[27,30]
					*Fusarium* sp.	[73]
					*Fusarium* spp.	[26]
				*Nectria*	*Nectria* sp.	[26]
			Sarocladiaceae	*Sarocladium*	*Sarocladium strictum* *=Acremonium strictum*	[24]
			/	*Tubercularia*	*Tubercularia* sp.	[26]
		Sordariales	Cephalothecaceae	*Phialemonium*	*Phialemonium dimorphosporum*	[24]
			Chaetomiaceae	*Chaetomium*	*Chaetomium* sp.	[26]
				*Mycothermus*	*Mycothermus thermophiles* *=Scytalidium thermophilum*	[30]
		Xylariales	Sporocadaceae	*Pestalotiopsis*	*Pestalotiopsis camelliae*	[24]
					*Pestalotiopsis fici*	[75]
					*Pestalotiopsis* sp.	[24,29]
					*Pestalotiopsis* spp.	[76]
			Xylariaceae	*Nemania*	*Nemania* sp.	[24]
			/	/	*Xylariales* sp.	[24]
Basidiomycota	Agaricomycetes	Agaricales	Schizophyllaceae	*Schizophyllum*	*Schizophyllum* sp.	[66]
		Russulales	Peniophoraceae	*Peniophora*	*Peniophora incarnata*	[24]
	/	/	/	/	*Uncultured Basidiomycota* sp.	[24]
Mucoromycota	Mucoromycetes	Mucorales	Mucoraceae	*Mucor*	*Mucor* sp.	[26]
				*Thamnidium*	*Thamnidium* sp.	[26]
3	6	14	24	34	Total number

**Table 3.** Endophytic bacteria isolated from tea plants.

Phylum	Class	Order	Family	Genus	Species	Reference
Actinobacteria	Actinobacteria	Micrococcales	Cellulomonadaceae	*Cellulomonas*	*Cellulomonas flavigena*	[38]
			Microbacteriaceae	*Microbacterium*	*Microbacterium arborescens*	[38]
					*Microbacterium imperiale*	[38]
					*Microbacterium testaceum*	[38]
		Streptomycetales	Streptomycetaceae	*Streptomyces*	*Streptomyces* sp.	[77]
Bacteroidetes	Bacteroidia	Bacteroidales	Prevotellaceae	*Prevotella*	*Prevotella loescheii*	[38]
	Flavobacteriia	Flavobacteriales	Flavobacteriaceae	*Myroides*	*Myroides* spp.	[35]
Firmicutes	Bacilli	Bacillales	Bacillaceae	*Bacillus*	*Bacillus amyloliquefaciens*	[52]
					*Bacillus safensis*	[40]
					*Bacillus subtilis*	[37]
					*Bacillus* sp.	[35]
					*Bacillus* spp.	[36]
				*Fictibacillus*	*Fictibacillus* spp.	[35]
				*Lysinibacillus*	*Lysinibacillus* spp.	[35]
				*Oceanobacillus*	*Oceanobacillus* spp.	[35]
			Paenibacillaceae	*Paenibacillus*	*Paenibacillus* spp.	[35]
			Planococcaceae	*Bhargavaea*	*Bhargavaea* spp.	[35]
			Staphylococcaceae	*Staphylococcus*	*Staphylococcus* spp.	[35]
Proteobacteria	Alphaproteobacteria	Caulobacterales	Caulobacteraceae	*Brevundimonas*	*Brevundimonas* sp.	[35]
		Rhizobiales	Bradyrhizobiaceae	*Bosea*	*Bosea* spp.	[35]
				*Bradyrhizobium*	*Bradyrhizobium* spp.	[35]
			Methylobacteriaceae	*Methylobacterium*	*Methylobacterium* spp.	[35]
			Rhizobiaceae	*Ensifer*	*Ensifer* spp.	[35]
		Rhodobacterales	Rhodobacteraceae	*Rhodobacter*	*Rhodobacter sphaeroides*	[35]
		Sphingomonadales	Sphingomonadaceae	*Sphingobium*	*Sphingobium* spp.	[35]
				*Sphingomonas*	*Sphingomonas* spp.	[35]
	Betaproteobacteria	Burkholderiales	Burkholderiaceae	*Burkholderia*	*Burkholderia cepacia*	[39]
					*Burkholderia* spp.	[35]
				*Ralstonia*	*Ralstonia* spp.	[35]
			Comamonadaceae	*Variovorax*	*Variovorax* spp.	[35]
			Oxalobacteraceae	*Herbaspirillum*	*Herbaspirillum* spp.	[35]
				*Massilia*	*Massilia* spp.	[35]
			/	*Aquincola*	*Aquincola* spp.	[35]
	Gammaproteobacteria	Enterobacterales	Erwiniaceae	*Pantoea*	*Pantoea* spp.	[35]
			Yersiniaceae	*Serratia*	*Serratia* spp.	[35]
		Pseudomonadales	Moraxellaceae	*Acinetobacter*	*Acinetobacter* spp.	[35]
			Pseudomonadaceae	*Pseudomonas*	*Pseudomonas fluorescens*	[38]
					*Pseudomonas* spp.	[35]
		Xanthomonadales	Rhodanobacteraceae	*Luteibacter*	*Luteibacter* spp.	[35,40]
			Xanthomonadaceae	*Stenotrophomonas*	*Stenotrophomonas* spp.	[77]
4	7	13	24	32	Total number	

‘/’ in the table means unclassified

## 4. Distribution of endophytic fungi and bacteria in tea plants and its influence factors

### 4.1. Distribution of endophytic fungi and bacteria in tea plants

The endophytic fungi and bacteria can be isolated from the roots, shoots, stems, leaves, and flowers of various cultivars of tea plants [50]. The number and types of endophytic fungi and bacteria in tea plants vary with the time of sample collection [26,35], tissue type, and cultivar of tea plants [26,35]. Different types of tea plant tissues have their own dominant microflora [35,50], and change with the season [35]. In addition, the distribution of the same flora in different parts of the plant is not evenly distributed and has specificity. The geographical environment of the tea plant has a certain influence on the number and types of endophytes.

### 4.2. Influencing factors of the distribution of endophytic fungi and bacteria in tea plants

#### 4.2.1. Effects of tissue types on the distribution of endophytic fungi and bacteria

The distribution of endophytic fungi and bacteria in tea plants has obvious tissue specificity. You *et al*. [26] isolated 143 endophytic fungi from the roots, stems and leaves of tea plants in the tea garden of Wutong Farm, Sichuan Province, China. Among them, 62 strains of fungi from 11 genera were isolated from the root, 55 strains of fungi 9 genera were isolated from the stem, and 36 strains of 6 genera were isolated from the leaves. Among them, the dominant fungal flora in roots, stems and leaves are *Fusarium* spp., *Trichoderma* sp. and *Alternaria* sp., respectively. Chen *et al*. [50]found that abundant endophytic fungi can be isolated from the leaves and branches of tea plants. The isolation frequency in mature leaves was as high as 93.88–100%. The isolation rate of endophytic fungi in petals, embryos, roots, buds and seed coats showed a clear downward trend. In the leaves of different developmental stages, the total isolation rate of endophytic fungi was higher, but the endophytic bacteria were dominant in the leaf buds and the isolation frequency was 65%, while the isolation frequency of endophytic fungi was 7.5%. In mature leaves, endophytic fungi were dominant and the separation frequency is 89.28%, but endophytic bacteria were not isolated. It shows that during the maturation of tea leaves, the endophytic bacteria in the leaves are gradually decreasing and the endophytic fungi are gradually increasing, showing the process of succession.

Win *et al*. [24] found in the tissue preference study of endophytic fungi in tea plants that *Colletotrichum gloeosporioides f*. sp. *camelliae* showed preference in bark and old leaf tissues rather than new leaf and xylem tissues. *Guignardia mangiferae* and *Glomerella* sp. were tissue-specific endophytic fungi, and they were only detected in leaf and stem, respectively. The above research indicated that there was a clear preference for tissue types of tea endophytic fungi and bacteria. As the maturity of the tissue increased, the dominant group of endophytic fungi and bacteria in the tissue will show a succession process.

**Figure 1.**
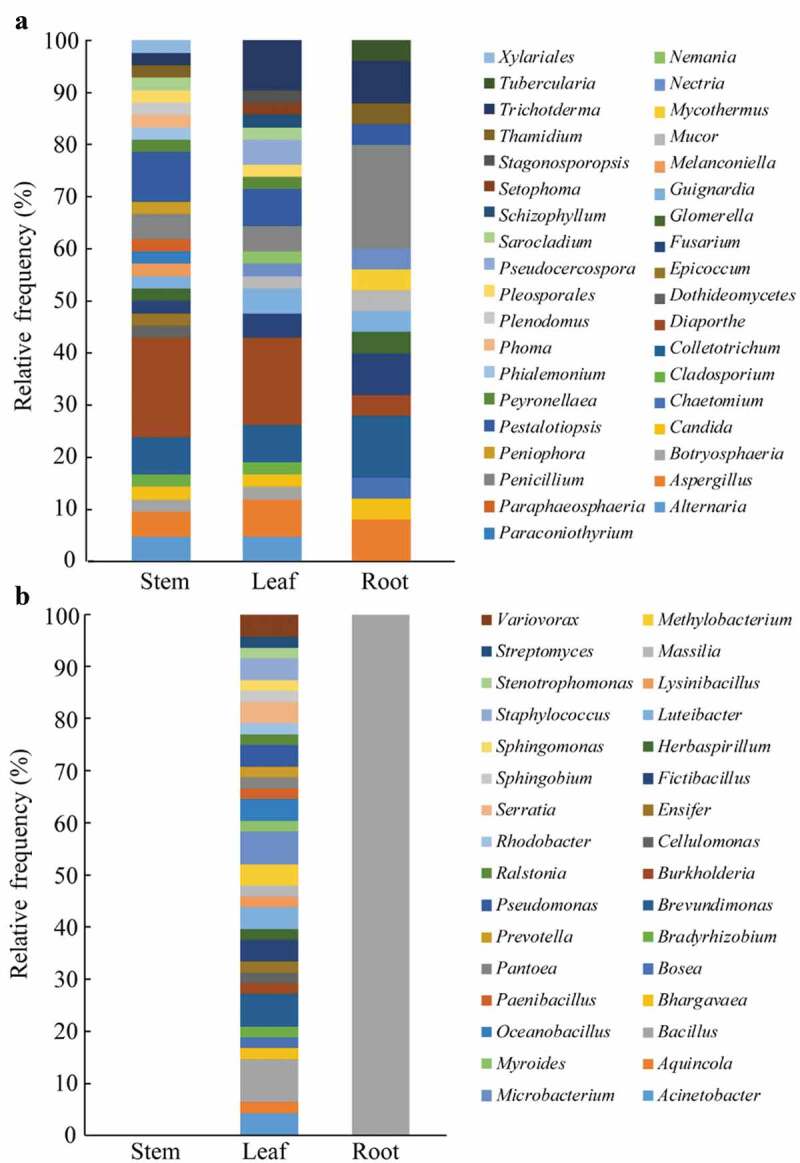
Distribution of tea endophytic fungi and bacteria (genus level) in different tissues. (a) Tea endophytic fungi in different tissues. (b) Tea endophytic bacteria in different tissues.

Figure 1 shows the distribution of endophytic fungi and endophytic bacteria in different tissues of tea plants. It can be seen that endophytic fungi show a preference for stem and leaf tissues. However, endophytic bacteria showed a preference for leaf tissues, but there are fewer endophytic bacteria in root and stem tissues. The reason for this result is that most studies only isolate tea endophytic bacteria from tea leaves, but there are fewer isolation experiments from root and stem tissues, so more in-depth research is needed to find out the distribution of tea endophytic bacteria.

#### 4.2.2. Effects of tea cultivars on the distribution of endophytic fungi and bacteria

The distribution of endophytic fungi and bacteria showed significant differences among different tea cultivars. Win *et al*. [24] isolated the endophytic fungi of three tea cultivars (Hokumei, Sayamakaori, and Yabukita) in the same tea garden and found that the four common endophytic fungi (*Pleosporales* sp., *Colletotrichum gloeosporioides f*. sp. *camelliae, Peyronellaea glomerata*, and *Botryosphaeria dothidea*) all showed obvious cultivars preference except *Pleosporales* sp. The endophytic bacterial communities that can be cultivated in the branches of Zijuan and Yunkang-10 tea cultivars with the same cultivation and management level under natural environment are quite different. These two tea cultivars contained the same 12 endophytic bacterial genera, but 18 genera were unique to one of the cultivars (Zijuan contained 7 genera and Yunkang-10 contained 11 genera) [35]. The physiological states of different tea cultivars are different, which will lead to differences in the distribution of endophytic fungi and bacteria, but the mechanism and specific relationship between the two need to be further studied.

#### 4.2.3 Effect of season on the distribution of endophytic fungi and bacteria

The physiological state of tea plants changes with the change of seasons, and the types and number of endophytic fungi and bacteria in the tea plants also change.

You *et al*. [26] isolated the endophytic fungi from the roots, stems and leaves of Zaobaijian-5 in March, May, July and September. It was found that the isolation rate of endophytic fungi was higher in March, May and September, but lower in July. Hu *et al*. [30] isolated endophytic fungi of the root from Longjing-changye cultivar in spring, summer and autumn. The dominant endophytic fungi in the root was *Glomerella cingulata*, while the isolation rate of *Glomerella cingulata* in spring and significantly different from that in summer and autumn. Yan *et al*. [35] isolated endophytic bacteria from the branches of Zijuan in Anhui Province tea garden during one year, and obtained 9, 70, 27 and 4 isolates in spring, summer, autumn and winter, respectively. The dominant endophytic bacteria group were *Variovorax* spp., *Herbaspirillum* spp. *Methylobacterium* spp. and *Bacillus* spp. respectively. The above results show that the number of endophytic fungi and bacteria in tea plants varies greatly with the seasons, and the dominant fungi and bacteria group also alternates with the seasonal changes.

## 5. Biological functions of tea endophytic fungi and bacteria

### 5.1. Antagonism with plant pathogens

**Figure 2.**
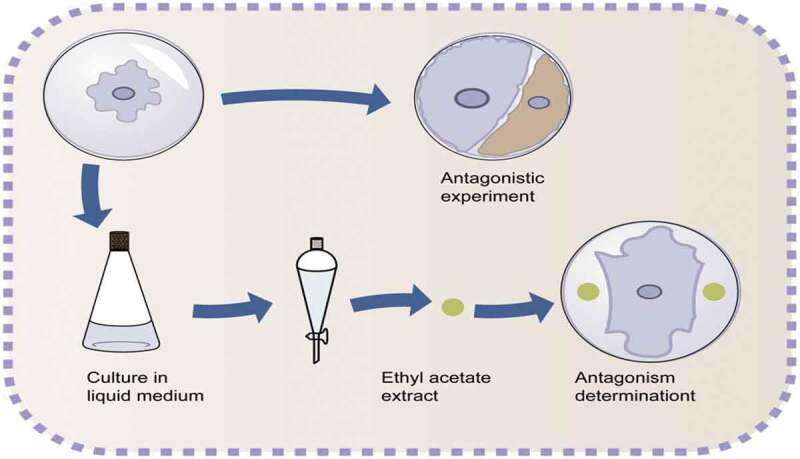
Antagonism with plant pathogens.

Plant pathogens are one of the main reasons leading to the reduction of crop yield and quality. There are more than 1900 fungi in the world that cause diseases in crops and pose a serious threat to food security [51]. Therefore, it is particularly important to prevent and control plant diseases. Some endophytic fungi and bacteria in tea plants have antagonistic effects against tea pathogens (Figure 2), so they have good biocontrol potential.

The strain *Bacillus subtilis* TL2 isolated from *Camellia sinensis* cv. *Tie-guanyin* leaves [36] had a strong inhibitory effect on the mycelial growth of four tea tree pathogens: *Phyllosticta gemmiphliae, Pestallozzia theae, Gloeosporium theae-sinensis* and *Neocapnodium theae*, the inhibition rate reached 83.6%, 83.3%, 90.3% and 86.5%, respectively. It can be seen that the tea endophytic bacteria has good application potential for the prevention and treatment of tea plant diseases. The strain *Colletotrichum gloeosporioides* CgloTINO1 isolated from the tea garden of Assam [32] in India had a strong antagonistic effect on the tea pathogens *Pestalotiopsis theae* and *Colletotrichum camelliae*. In further research, it was found that the cell-free culture filtrate of this strain also showed high antagonistic activity against both the pathogens. The cell-free culture filtrate of the 5-day-old cultures had a higher inhibitory effect than the 20-day-old cultures. The chitinase and protease activities in the two cultures were significantly different, indicating that these enzymes played a role in the process of antagonism. The study explained the antagonism of tea endophytic fungi and plant pathogens from the perspective of enzymes. However, some secondary metabolites of fungi also have a certain effect, which can be further studied.

*Pantoea ananatis* is a kind of bacteria attached to the leaves of tea plants, which can induce or aggravate the occurrence of frost damage in tea plants. The strain *Bacillus amyloliquefaciens* Y1 isolated from the healthy tissues of tea plants by Xiaoqin Huang has a significant inhibitory effect on the colony expansion of *Pantoea ananatis* [52]. Many tea gardens in China have suffered large losses due to the freezing damage caused by the cold spring. The results of this study can provide an idea for preventing and controlling frost damage in tea plants.

In addition to the antagonism of tea plant pathogens, tea endophytic fungi also have antagonistic effects on other plant pathogens. The strain *Rhizobium radiobacter* EB659 isolated from the leaves of Fuyunliuhao (a tea cultivar) can effectively inhibit tomato pathogenic bacteria (*Ralstonia solanacearum*), watermelon pathogenic fungi (*Acidovoraxavenaesub* sp. *citrulli*) and cotton pathogenic fungi (*Verticillium dahliae*), especially on *Acidovoraxavenaesub* sp. *citrulli* [38]. The endophytic fungi *Pseudocercospora kaki* and *Penicillium sclerotiorum* isolated from the leaves of the tea plant had obvious inhibitory effects on rice blast pathogen *Magnaporthe grisea*, and their dual-culture broth and ethyl acetate extracts have significantly stronger inhibitory effects on the rice blast pathogen *Magnaporthe grisea* than single culture broth [53]. The above results indicate that a variety of endophytic fungi synergistically antagonize the pathogen is a more effective way. It is reported that the volatile compounds produced by *Burkholderia tropica* can inhibit the growth of pathogenic fungi [54], while the research on the inhibition of tea endophytic fungi and bacteria to plant pathogens is less, which needs further study.

### 5.2. Plant-growth promoting activities

**Figure 3.**
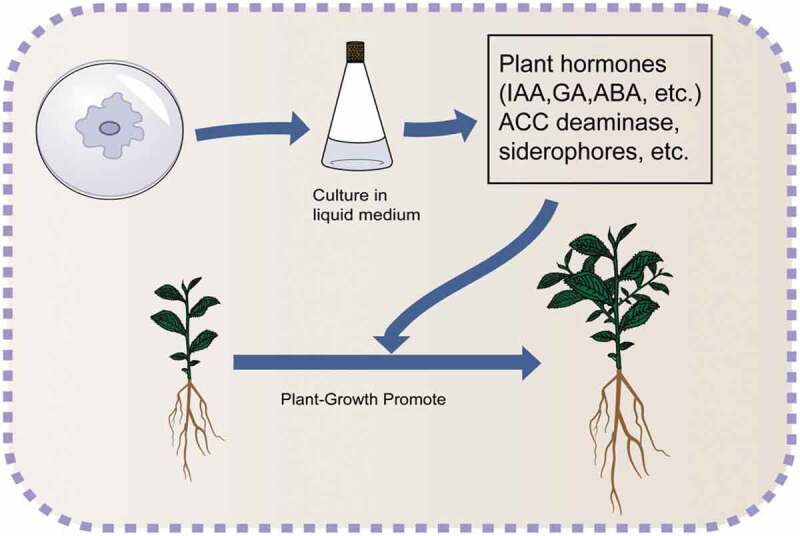
Plant-growth promoting activities.

Some endophytic fungi and bacteria promote growth of plants by providing indoleacetic acid, gibberellin, cytokinin, siderophores, phosphate-solubilizing enzymes and ACC deaminase (Figure 3) [4].

Yan *et al*. [35] tested plant-growth-promoting (PGP) activities of endophytic bacteria obtained from two tea cultivars of Zijuan and Yunkang-10, and found that the *Herbaspirillum* spp., *Methylobacterium* spp., and *Brevundimonas* spp. showed PGP abilities. The endophytic bacteria *Burkholderia cepacia* G3 strain isolated from the tea plant had significant PGP abilities. Moreover, the strain also has a significant promotion effect on wheat seed germination and seedling growth [39]. NATH *et al*. [27] evaluated the PGP activities of endophytic fungi isolated from various parts of tea plants, and found that among these endophytic fungi, *Aspergillus niger* has the best potassium solubilizing ability and IAA-producing ability. *Fusarium oxysporum* has the best GA3 producing ability and *Penicillium sclerotiorum* has the best zinc solubilizing ability. The above research shows that some endophytic fungi and bacteria in tea plants have significant PGP capacity, so they are a potential resource that can be developed into an efficient biological fertilizer.

### 5.3. Capacity of producing bioactive metabolites

Tea endophytic fungi can produce some biologically active products during the cultivation process. Wang *et al*. [55] isolated nine compounds from the solid culture of the endophytic fungi *Alternaria alternata* obtained from fresh shoots of tea plants. Among these compounds, alternariol had the strongest inhibitory effect on *Bacillus subtilis*, MIC_80_ was 8.6 μg/ml. And alternolol 9-methyl ether had a slight cytotoxic effect on human osteosarcoma cells U2OS, with IC_50_ of 28.3 μM. The other seven metabolites have moderate to slight inhibitory effects on the pathogenic microorganisms in the test. GC-MS analysis of the culture broths of endophytic fungi *Pseudocercospora kaki* and *Penicillium sclerotiorum* from tea plants revealed that there were more biologically active substances in the dual-culture broth than in the single broth [53]. This reveals the reason why the inhibition effect of dual-culture broth on *Magnaporthe grisea* is better than that of single culture broth.

The ability of endophytes in tea plants to produce metabolites with biological activity has great potential for application against plant pathogenic bacteria and can be used as a resource for fungi or bacterial inhibitors. Extreme environments can stimulate fungi to produce new metabolites. Although some metabolites do not directly participate in the basic metabolic processes of growth and energy generation, they are beneficial to their survival in extreme environments [56,57]. This provides guidance for the development of valuable metabolites of tea endophytes.

### 5.4. Produce and modify tea plant metabolites

**Figure 4.**
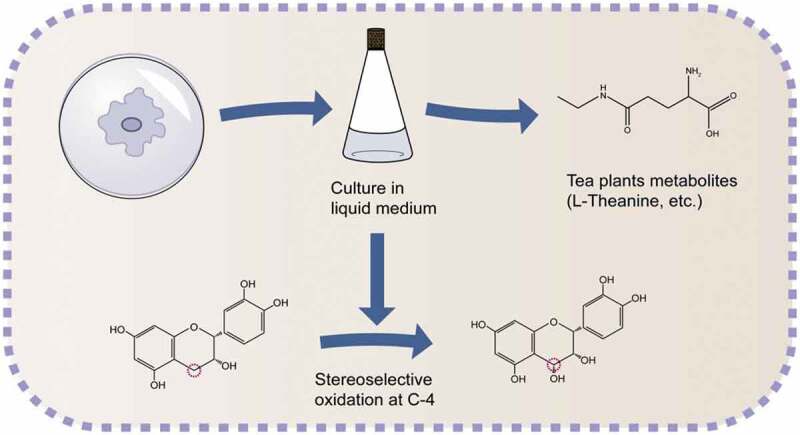
Produce and modify tea plant metabolites.

Tea endophytes can modify the structure of some metabolites in tea plants (Figure 4). The endophytic fungus *Diaporthe* sp. isolated from tea plants can stereoselectively oxidize the C-4 carbon of 2 R-substituted flavans to 3-hydroxy structure from the same direction [58]. The endophytic bacteria *Luteibacter* spp. isolated from tea plant can produce theanine, which is the highest nonprotein amino acid in tea plant and plays an important role in the quality of tea [40]. Endophytic fungi and bacteria can produce or modify the metabolites of tea plants, which can provide a new way to study the interaction between endophytes and plant hosts. In addition, it can provide ideas for enriching the content of tea leaves to improve the quality of tea.

### 5.5. Other biological functions

In addition to the above functions, tea endophytic fungi and bacteria have many other functions. In addition to being antagonistic to pathogens, the tea endophytic bacteria *Bacillus subtilis* TL2 strain exhibits a strong ability to degrade cypermethrin, which provides a new method for disease prevention and pesticide residue management [36]. Enzymes are part of the natural metabolism of all living organisms. They are biocatalysts that increase the rate of metabolic reactions [59]. Zhang *et al*. [60] isolated many endophytic fungi that can produce polyphenol oxidase from tea plants. The polyphenol oxidase can effectively catalyze the oxidation of polyphenols to quinones, and it has been well researched and applied in the fields of phenol-containing wastewater treatment, lignin degradation and dye decolorization [61].

## 6. Conclusions and future insights

Endophytic fungi and bacteria are a new type of microbial resources, which have great research value and broad development prospects. Plant diseases affect crops all over the world, resulting in a global food production decline of about 10% [62]. Currently, pesticides are mainly used to reduce losses in agricultural production [63]. However, the heavy use of pesticides will adversely affect human and ecosystem functions, such as pollution to the environment, food chain and soil [64], reducing soil fertility and breaking the ecological balance, which threatens the sustainable development of agriculture [65]. Biocontrol is one of the ways to control the occurrence of plant diseases and has the least impact on the environment [66]. The biological control agents or products include macroorganisms, microorganisms, chemical mediators, and natural substances [67]. Some endophytes also have antagonistic effects against plant pathogens. Larran *et al*. [66] demonstrated in a study on wheat that wheat endophytic fungi can significantly reduce tan spot of wheat caused by *Drechslera tritici-repentis*. At present, through dual-culture experiments, it has been found that some endophytic fungi and bacteria from tea plants have antagonistic effects on tea plant pathogens, but further research on the mechanism and effects in practical applications is needed to provide new ways for biocontrol of tea plant diseases. The endophytic fungi and bacteria in tea plants have an inhibitory effect on the growth of pathogens of other crops such as wheat and watermelon. Therefore, tea endophytic fungi and bacteria have good potential for biocontrol and are environmentally friendly.

Endophytic fungi and bacteria metabolism produce abundant natural compounds, such as antimicrobial metabolites, high-value host plant secondary metabolites and other medicinal metabolites [18], tea plant is also a medicinal plant, including tea polyphenols, catechins, theanine and other medicinal ingredients, but only endophytic bacteria that can produce theanine have been reported. At the same time, there are few studies on the functions of secondary metabolites of tea endophytic fungi and bacteria. In addition, tea endophytic fungi can modify the secondary metabolites in tea plants. These functions will have an impact on the host’s physiological metabolism, so further study of their interaction mechanism and actual effect can provide a new way to improve crop quality.

Endophytic fungi and bacteria can produce plant hormones, siderophores, phosphate-solubilizing enzymes (ACC), deaminase and enhance the absorption of soil nutrients such as nitrogen, phosphorus, and other nutrients by the host plant to achieve the function of promoting plant growth [68,69]. Some tea endophytic fungi and bacteria have plant growth-promoting functions. In the future, the actual promotion effect of tea tree growth should be thoroughly explored to provide a new way to increase tea production. With the widespread use of plant hormones in agriculture, the microbial production of plant hormones will have bright prospects [70] and tea endophytic fungi and bacteria will also become potential resources for the production of plant hormones. In addition, some microorganisms produce siderophores that cannot be used by pathogenic microorganisms to inhibit their growth and reduce the occurrence of plant diseases [71]. The siderophores production characteristics of tea endophytic fungi and bacteria in the process of plant growth promotion also make it have biocontrol potential.

In general, the current research on tea endophytic fungi and bacteria is relatively shallow, so more in-depth research is needed to clarify the interaction mechanism between endophytic fungi and bacteria and tea plants, so as to provide a better understanding on ecological and medical implications of tea plants-endophytic microbiota interaction and exploitable resources.

## Highlights

- There are abundant endophytic fungi and bacteria in tea plants.
- Endophytic microbiota enhances the resistance of tea plant to stresses.
- Metabolites produced by microbiota from tea plant are important bioresource.
- Microbiota assembly mechanisms and their biological functions need more exploration.
